# A role for chromosomal instability in the development of and selection for radioresistant cell variants

**DOI:** 10.1054/bjoc.2000.1604

**Published:** 2001-02

**Authors:** C L Limoli, J J Corcoran, R Jordan, W F Morgan, J L Schwartz

**Affiliations:** Department of Radiation Oncology, University of California, San Francisco, CA 94103–0806, USA; Department of Radiation Oncology, University of Maryland School of Medicine, Baltimore, MD, 21201, USA; Department of Radiation Oncology, University of Washington, Seattle, WA, 98195, USA

**Keywords:** chromosome instability, radioresistance, DNA repair, radiation therapy

## Abstract

Chromosome instability is a common occurrence in tumour cells. We examined the hypothesis that the elevated rate of mutation formation in unstable cells can lead to the development of clones of cells that are resistant to the cancer therapy. To test this hypothesis, we compared chromosome instability to radiation sensitivity in 30 independently isolated clones of GM10115 human–hamster hybrid cells. There was a broader distribution of radiosensitivity and a higher mean SF _2_ in chromosomally unstable clones. Cytogenetic and DNA double-strand break rejoining assays suggest that sensitivity was a function of DNA repair efficiency. In the unstable population, the more radioresistant clones also had significantly lower plating efficiencies. These observations suggest that chromosome instability in GM10115 cells can lead to the development of cell variants that are more resistant to radiation. In addition, these results suggest that the process of chromosome breakage and recombination that accompanies chromosome instability might provide some selective pressure for more radioresistant variants. © 2001 Cancer Research Campaign http://www.bjcancer.com


*British Journal of Cancer* (2001) **84**(4), 489–492
© 2001 Cancer Research Campaign
doi: 10.1054/ bjoc.2000.1604, available online at http://www.idealibrary.com on IDEAL®

http://www.bjcancer.com

### A role for chromosomal instability in the development of and selection for radioresistant cell variants

**CL Limoli[1], JJ Corcoran[2], R Jordan[3], WF Morgan[2] and JL Schwartz[3]**

[1]Department of Radiation Oncology, University of California, San Francisco, CA 94103–0806, USA; [2]Department of Radiation Oncology, University of Maryland School of Medicine, Baltimore, MD 21201, USA; [3]Department of Radiation Oncology, University of Washington, Seattle, WA 98195, USA

**Summary** Chromosome instability is a common occurrence in tumour cells. We examined the hypothesis that the elevated rate of mutation formation in unstable cells can lead to the development of clones of cells that are resistant to the cancer therapy. To test this hypothesis, we compared chromosome instability to radiation sensitivity in 30 independently isolated clones of GM10115 human–hamster hybrid cells. There was a broader distribution of radiosensitivity and a higher mean $SF_2$ in chromosomally unstable clones. Cytogenetic and DNA double-strand break rejoining assays suggest that sensitivity was a function of DNA repair efficiency. In the unstable population, the more radioresistant clones also had significantly lower plating efficiencies. These observations suggest that chromosome instability in GM10115 cells can lead to the development of cell variants that are more resistant to radiation. In addition, these results suggest that the process of chromosome breakage and recombination that accompanies chromosome instability might provide some selective pressure for more radioresistant variants. © 2001 Cancer Research Campaign http://www.bjcancer.com

**Keywords**: chromosome instability; radioresistance; DNA repair; radiation therapy

It is well established that there are wide variations in the inherent radiation sensitivity of tumour cells. These variations in radiosensitivity play an important role in tumour response to radiation therapy; tumours that contain more radioresistant cells do not respond as well to therapy (West, 1994). There are many factors that contribute to variations in sensitivity (West, 1994; Szumiel, 1981). Some of the variations in radiation sensitivity reflect the sensitivity of the tissue from which the tumour developed (Fertil and Malaise, 1985; Weichselbaum et al, 1989). Thus cells from squamous cell carcinomas tend to be more resistant to radiation than cells from soft tissue sarcomas. Some of the variation in radiation sensitivity is due to the inherent sensitivity of the individual; there are correlations between tumour cell radiosensitivity and normal tissue radiosensitivity from the same individual (Dahlberg et al, 1993; West et al, 1998). There are also tumour-specific factors that influence radiation sensitivity. For example, alterations in certain oncogenes (Kasid et al, 1987; Sklar, 1988; McKenna et al, 1990), loss or attenuation in cell cycle checkpoint control (McKenna et al, 1991; Bristow et al, 1996), and DNA ploidy changes (Schwartz et al, 1999) all common to many tumours, can change radiation responses.

As radiation sensitivity is a complex phenotype that is influenced by many different factors, another feature of tumour cells that may contribute to variations in radiation sensitivity is genome instability. Genomic instability is defined as an increase in the rate of acquisition of alterations in the mammalian genome. Genomic instability is a common feature of tumours (Mitelman, 1991; Solomon et al, 1991; Rabbits, 1994). It can also be induced or significantly increased by radiation exposure (Morgan et al, 1996). The increased rates of mutation in unstable cells may increase the probability of changing one of these factors and thereby changing radiation sensitivity. Thus one would predict greater variations in radiation sensitivity in genomically unstable clones of cells as compared to stable clones. To test this hypothesis we examined radiation sensitivity in a series of clonally expanded Chinese hamster ovary (CHO) cells characterized as either chromosomally stable or unstable. We observed that instability was associated with a broader distribution of radiation sensitivities as predicted, but that most of the increased variability was due to the presence of more radioresistant clones.

*Received 6 April 2000*
*Revised 19 October 2000*
*Accepted 27 October 2000*

*Correspondence to:* JL Schwartz

### MATERIALS AND METHODS

All subclones originated from the parental GM10115 cell line, a human CHO hybrid containing one copy of human chromosome 4 in a background of 22–24 hamster chromosomes. Cells were maintained as log-phase cultures in Dulbecco's modified Eagle's medium supplemented with 10% fetal bovine serum, 2 mM L-glutamine, 100 U $ml^{-1}$ of penicillin, 100 mg $ml^{-1}$ of streptomycin, and 0.2 mM L-proline. Cells were cultured at 34˚C in humidified incubators containing 5% $CO_2$ in air, where routine doubling times of 24–27 h were obtained.

Subclones were derived and expanded from single progenitor cells from either unirradiated cells or from cells surviving a previous exposure to 10 Gy of X-rays. The status of chromosomal stability for each clone was established as previously described (Limoli et al, 1997, 1998; Ponnaiya et al, 1998). Minimums of 200 metaphases were scored for each sample, and only those rearrangements involving the human chromosome were scored. Chromosomal instability was defined operationally to include any clone derived from a single cell that shows at least three distinct metaphase subpopulations involving rearrangements of the human chromosome, in which all such rearrangements account for a minimum of 5% of the total metaphases scored. Analysis of these subclones has indicated their capacity to maintain the phenotype of chromosomal instability over multiple (> 80) generations. Chromosome instability was confirmed in each clone before any other analyses.

For survival measurements, exponentially growing cultures were exposed to X-rays at 2.5 Gy min$^{-1}$ using a Phillips RT250 X-ray machine (250-kV peak, 15 mA; half-value layer 1.0 mm copper). Immediately following irradiation cells were diluted and plated into 100 mm dishes containing 15 ml of medium to determine the surviving fraction by clonogenic assay. After 1–2 weeks of growth, plates containing visible colonies of ≥50 cells were stained with 0.1% crystal violet in 25% ethanol and counted. All survival measurements were corrected for plating efficiency. Plating efficiency was determined by plating 100 cells per dish in triplicate, while the surviving fraction measured after 2.0 Gy of X-rays ($SF_2$) was determined by plating 200 cells per dish in quintuplicate.

DNA double-strand break rejoining proficiency was measured in the GM10115 cells using pulsed field gel electrophoresis (PFGE) to estimate break frequencies following irradiation (Schwartz et al, 1995). Cells were exposed to 50 Gy X-rays (6 Me V) in ice-cold phosphate-buffered saline (PBS) and either immediately sampled or held 1 h in complete medium at room temperature to measure break rejoining. DNA was run on CHEF-DR III apparatus (BioRad). The PFGE parameters were 18 h run at 2 V cm$^{-1}$ with an initial and final switch time of 1800 s and an included angle of 108˚. Following electrophoresis the gel was air-dried overnight and exposed to a storage phosphor screen (Kodak). The percentage of DNA entering the gel was determined by densitometry. These measurements were used to calculate relative break frequency and the percentage of breaks rejoined in 1 h. Results are expressed as the percentage of breaks rejoined in 1 h ± SEM for at least 3 experiments.

$G_2$ chromosome radiosensitivity was measured by exposing exponentially growing cultures to 1.5 Gy of $^{137}$Cs gamma rays. The cultures were then incubated at 37˚C for a further 30 min before 0.2 μM colcemid was added to the cultures and cells harvested 1 h later by standard methods (Schwartz et al, 1999). Slides are air-dried and stained with a 2% Giemsa in Gurr buffer solution. 25 cells per experiment were analysed for chromatid-type aberrations. Chromatid-type aberrations are classified as chromatid (and isochromatid) deletions or chromatid exchanges. Deletions were distinguished from gaps by displacement of the chromatids. At least 3 independent determinations were made for each cell clone. Correlation and statistical significance between groups of stable and unstable subclones were assessed by regression analysis, analysis of variance (ANOVA), and one and two tailed student *t*-tests.

### RESULTS

Clonogenic assay in 10 previously unirradiated control clones, 10 irradiated but chromosomally stable clones, and 10 irradiated and chromosomally unstable clones determined radiation sensitivity. The results are shown in Figure 1. There was a narrow distribution of radiosensitivity for the stable clones. $SF_2$ ranged from 0.25–0.46. The mean $SF_2$ was 0.36 ± 0.02. These values are comparable to the range of sensitivities (0.21–0.44) and mean (0.34 ± 0.02) $SF_2$ seen in 10 random control clones isolated from an unirradiated population of GM10115 cells. In contrast to the stable clones, the range of radiosensitivities for the unstable clones was broader (0.23–0.67) and the mean $SF_2$ at 0.46 ± 0.04 was significantly greater than that for the stable clones ($P = 0.034$, one-tailed *t*-test). There appeared to be two clusters of radiation sensitivities in the unstable clones. 3 of the unstable clones had radiation sensitivities that were similar to those of the stable clones. 7 of the unstable clones had significantly higher $SF_2$s ($P < 0.007$). Thus chromosome instability was associated with greater variation in radiation sensitivity and a shift in the population to greater radioresistance.

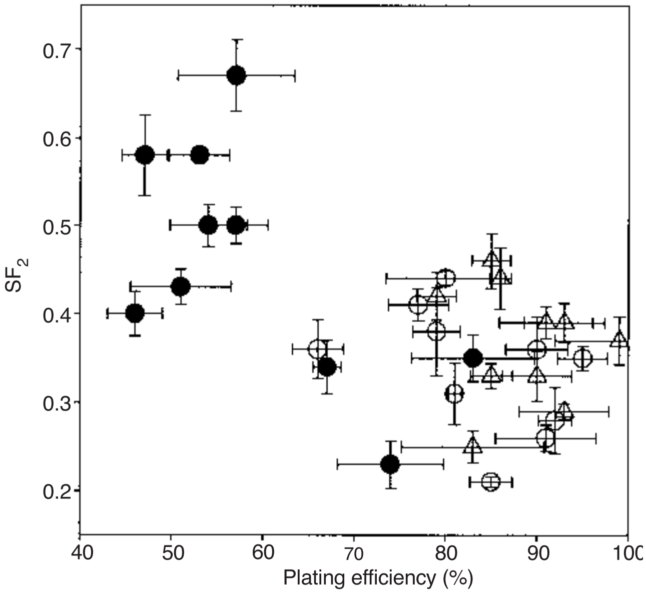

**Figure 1** Plating efficiency (%) and radiosensitivity ($SF_2$) measured in stable (○), unstable (●), and previously unirradiated (△) GM10115 control subclones. Mean and SEM of 3 independent determinations

The mean plating efficiency (84 ± 8.7%) measured for the 10 random control clones isolated from unirradiated GM10115 cells was similar to the 88 ± 6% plating efficiency measured in the irradiated but chromosomally stable clones (Figure 1). As has been previously noted (Chang and Little, 1991; Limoli et al, 1997; Mothersill and Seymour, 1997, unstable clones have significantly ($P<0.001$) lower plating efficiencies (59 ± 12%) as compared to stable clones (88 ± 6%). In the unstable population, the more radioresistant clones also had the lowest plating efficiencies as compared to more sensitive clones ($P < 0.0004$).

DNA repair characteristics were determined by two assays. The first was a cytogenetic assay where chromosome aberrations induced in $G_2$ were analysed in 7 clones of varying radiosensitivity (Figure 2A). The relationship between chromosome aberration induction and $SF_2$ was biphasic. The two most radiosensitive clones had the highest aberration frequencies. The other four clones had similar aberration frequencies even though they ranged in sensitivities from 0.3 to 0.6. There was no difference between stable and unstable clones in their response to this assay.

We next measured DNA double-strand break rejoining by PFGE in 8 clones with variable radiosensitivity (Figure 2B). While the relationship between break rejoining and radiation sensitivity was not significant ($P = 0.064$), there was clearly a trend in the data with the more resistant clones rejoining more breaks in 1 h. As with the cytogenetic assay, there was no difference between

© 2001 Cancer Research Campaign

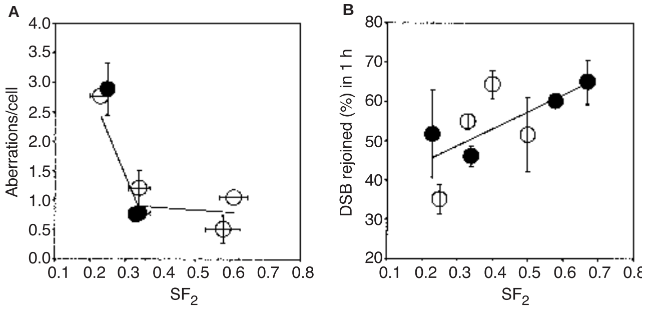

**Figure 2** Relationship between $SF_2$ and (**A**) $G_2$ chromosome break induction or (**B**) percentage of breaks rejoined in 1 h measured in stable (○) and unstable (●) clones. Mean and SEM of at least 3 experiments are presented stable and unstable clones in their relationship between breaks rejoined and radiosensitivity.

### DISCUSSION

The induction of genomic instability is considered an important prerequisite for oncogenesis (Nowell, 1976; Loeb, 1991). Genomic instability is also a common feature of tumours (Mitelman, 1991; Solomon et al, 1991; Rabbits, 1994), and it can be induced or significantly increased by exposure to cytotoxic agents such as ionizing radiation (Morgan et al, 1996). It has been suggested that genome instability may play an important role in tumour response to therapy and in particular in the development of resistance to therapy (Schimke et al, 1984; Morgan and Murnane, 1995). Given the complexity of factors that affect tumour sensitivity to cytotoxic cancer therapy, the elevated mutation rates associated with genomic instability could lead to the development of tumour cell variants that are more resistant to the therapy. Under the selective pressure of treatment, these more resistant clones might expand and ultimately contribute to therapy failure. Our results support this hypothesis. We observed greater variations in radiation sensitivity in the unstable versus stable clones (Figure 1). However, our results also suggest that the process that induces or perpetuates chromosomal instability provide some selection for more radioresistant variants.

There was clearly a shift in the distribution of radiosensitivities to more resistant cells. The mean $SF_2$ for the unstable clones was significantly larger than that for the unstable clones. Furthermore, this difference was not due to an inherent variability in the GM10115 population or the prior radiation exposure, as control subclones from both previously unirradiated and irradiated cells were nearly identical to each other in both plating efficiency and $SF_2$. The resistance phenotype that developed in the unstable clones was also not a consequence of alterations in cell growth rate, distribution of cells in the cell cycle, or variations in ploidy. The values for all 3 endpoints were similar in stable and unstable clones, and were not related to radiation sensitivity (unpublished observations).

The chromosome instability seen in these GM10115 cells manifests itself as increases in dicentric chromosomes and translocations (Marder and Morgan, 1993; Kaplan et al, 1997; Limoli et al, 1997). These alterations imply high spontaneous rates of chromosome breakage and recombination. This high level of breakage probably underlies the lower plating efficiencies in the unstable clones (Figure 1). We suggest that the high levels of chromosome breakage and recombination cycles in unstable clones may act to select for clones that are more efficient at break recombination and thus are more radioresistant. In support of this hypothesis are the observations that the more resistant clones tend to be more efficient at rejoining breaks as measured by cytogenetic and PFGE assays (Figure 2). The relationship between radiation sensitivity and repair as measured by either the G2 chromosome assay of PFGE, suggests that there may be different mechanisms underlying the resistance as there was no simple linear relationship between break rejoining and $SF_2$ for either assay.

Our observation that chromosome instability is associated with greater variation in radiation sensitivity and a greater likelihood of the development of radioresistant cell variants suggests that chromosome instability may have an important influence on the success of failure of radiotherapy. Tumours with higher levels of instability may be more difficult to control with standard courses of therapy because they are more prone to develop resistant subpopulations of cells. The ability of radiation at doses used in standard fractionation protocols (Limoli et al, 1999) to induce instability suggests that even in the absence of a prior instability, resistance can develop subsequent to the induction of instability. Standard fractionation procedures may exacerbate the potential problem by providing additional selection for radioresistant variants. The consequences of radiation treatment may therefore be more extensive than previously realized, and future directions in radiotherapy may require new approaches to minimize the potential detrimental effects of radiation-induced genomic instability.

### ACKNOWLEDGEMENTS

This work was supported by grant CA 73924 (C.L.L. and W.F.M.) and CA 73931 (J.L.S. and R.J.) from NCI and NASA, and NIH grant GM 54189 (W.F.M.). We thank Brian Ponnaiya for useful discussions, Erich Giedzinski for technical assistance, Karen Singer, Elizabeth Miller, and Mark Phillips for their help with dosimetry and radiation treatment. The data presented in this manuscript were presented by W.F.M. at the 42nd Annual Meeting of the American Society for Therapeutic Radiology and Oncology (ASTRO) Boston, October 22–26, 2000.

### REFERENCES

Bristow RG, Benchimol S and Hill RP (1996) The p53 gene as a modifier of intrinsic radiosensitivity: implications for radiotherapy. *Radiother Oncol* **40**: 197–223

Chang WP and Little JB (1991) Delayed reproductive death in X-irradiated Chinese hamster ovary cells. *Int J Radiat Biol* **60**: 483–496

Dahlberg WK, Little JB, Fletcher JA, Suit HD and Okunieff P (1993) Radiosensitivity in vitro of human soft tissue sarcoma cell lines and skin fibroblasts derived from the same patients. *Int J Radiat Biol* **63**: 191–198

Fertil B and Malaise EP (1985) Intrinsic radiosensitivity of human cell lines is correlated with radioresponsiveness of human tumors: analysis of 101 published survival curves. *Int J Radiat Oncol Biol Phys* **11**: 1699–1707

Kaplan MI, Limoli CL and Morgan WF (1997) Perpetuating radiation-induced chromosomal instability. *Radiat Oncol Investig* **5**: 124–128

Kasid U, Pfeifer A, Weichselbaum RR, Dritschilo A and Mark GE (1987) The raf oncogene is associated with a radiation-resistant human laryngeal cancer. *Science* **237**: 1039–1041

Limoli CL, Kaplan MI, Corcoran J, Meyers M, Boothman DA and Morgan WF (1997) Chromosomal instability and its relationship to other end points of genomic instability. *Cancer Res* **57**: 5557–5563

Limoli CL, Hartmann A, Shephard L, Yang CR, Boothman DA, Bartholomew J and Morgan WF (1998) Apoptosis, reproductive failure, and oxidative stress in Chinese hamster ovary cells with compromised genomic integrity. *Cancer Res* **58**: 3712–3718

© 2001 Cancer Research Campaign 

Limoli CL, Corcoran JJ, Milligan JR, Ward JF and Morgan WF (1999) Critical target and dose and dose-rate responses for the induction of chromosomal instability by ionizing radiation. *Radiat Res* **151**: 677–685

Loeb LA (1991) Mutator phenotype may be required for multistage carcinogenesis. *Cancer Res* **51**: 3075–3079

Marder BA and Morgan WF (1993) Delayed chromosomal instability induced by DNA damage. *Mol Cell Biol* **13**: 6667–6677

McKenna WG, Weiss MC, Bakanauskas VJ, Sandler H, Kelsten ML, Biaglow J, Tuttle SW, Endlich B, Ling CC and Muschel RJ (1990) The role of the H-ras oncogene in radiation resistance and metastasis. *Int J Radiat Oncol Biol Phys* **18**: 849–859

McKenna WG, Iliakis G, Weiss MC, Bernhard EJ and Muschel RJ (1991) Increased G2 delay in radiation-resistant cells obtained by transformation of primary rat embryo cells with the oncogenes H-ras and v-myc. *Radiat Res* **125**: 283–287

Mitelman F (1991) *Catalog of Chromosome Aberrations in Cancer, 4th Edition*. Wiley-Liss: New York

Morgan WF and Murnane JP (1995) A role for genomic instability in cellular radioresistance? *Cancer Metastasis Rev* **14**: 49–58

Morgan WF, Day JP, Kaplan MI, McGhee EM and Limoli CL (1996) Genomic instability induced by ionizing radiation. *Radiat Res* **146**: 247–258

Mothersill C and Seymour C (1997) Lethal mutations and genomic instability. *Int J Radiat Biol* **71**: 751–758

Nowell PC (1976) The clonal evolution of tumor cell populations. *Science* **194**: 23–28

Ponnaiya B, Limoli CL, Corcoran J, Kaplan MI, Hartmann A and Morgan WF (1998) The evolution of chromosomal instability in Chinese hamster cells: a changing picture? *Int J Radiat Biol* **74**: 765–770

Rabbitts TH (1994) Chromosomal translocations in human cancer. *Nature* **372**: 143–149

Schimke RT, Beverley S, Brown P, Cassin R, Federspiel N, Gasser C, Hill A, Johnston R, Mariani B, Mosse E, et al. (1984) Gene amplification and drug resistance in cultured animal cells. *Cancer Treat Rev* **11 Suppl A**: 9–17

Schwartz JL, Brinkman WJ, Kasten L, Miller DW, Moan EI, Murphy YT, Stella D and Sedita BA (1995) Altered metaphase chromosome structure in xrs-5 cells is not related to its radiation sensitivity or defective DNA break rejoining. *Mutat Res* **328**: 119–126

Schwartz JL, Murnane J and Weichselbaum RR (1999) The contribution of DNA ploidy to radiation sensitivity in human tumour cell lines. *Br J Cancer* **79**: 744–747

Sklar MD (1988) The ras oncogenes increase the intrinsic resistance of NIH 3T3 cells to ionizing radiation. *Science* **239**: 645–647

Solomon E, Borrow J and Goddard AD (1991) Chromosome aberrations and cancer. *Science* **254**: 1153–1160

Szumiel I (1981) Intrinsic radiosensitivity of proliferating mammalian cells. *Adv Radiat Biol* **9**: 281–322

Weichselbaum RR, Rotmensch J, Ahmed-Swan S and Beckett MA (1989) Radiobiological characterization of 53 human tumor cell lines. *Int J Radiat Biol* **56**: 553–560

West CML (1994) Predictive assays in radiation therapy. *Adv Radiat Biol* **18**: 149–180

West CM, Davidson SE, Elyan SA, Swindell R, Roberts SA, Orton CJ, Coyle CA, Valentine H, Wilks DP, Hunter RD and Hendry JH (1998) The intrinsic radiosensitivity of normal and tumor cells. *Int J Radiat Biol* **73**: 409–413

*© 2001 Cancer Research Campaign*
